# A tropism-transformed Oncolytic Adenovirus with Dual Capsid Modifications for enhanced Glioblastoma Therapy

**DOI:** 10.7150/jca.46463

**Published:** 2020-07-29

**Authors:** Lizheng Wang, Wenmo Liu, Zhe Li, Xupu Wang, Xinyao Feng, Zixuan Wang, Jiaxin Wu, Haihong Zhang, Hui Wu, Wei Kong, Bin Yu, Xianghui Yu

**Affiliations:** 1 National Engineering Laboratory for AIDS Vaccine, School of Life Sciences, Jilin University, Changchun 130012, China.; 2 Key Laboratory for Molecular Enzymology and Engineering, the Ministry of Education, School of Life Sciences, Jilin University, Changchun 130012, China.

**Keywords:** Glioblastoma, oncolytic adenovirus, TRAIL, Ad37, fiber knob

## Abstract

Glioblastoma, the most common human brain tumor, is highly invasive and difficult to cure using conventional cancer therapies. As an alternative, adenovirus-mediated virotherapies represent a popular and maturing technology. However, the cell surface coxsackievirus and adenovirus receptor (CAR)-dependent infection mechanism limits the infectivity and oncolytic effects of Adenovirus type 5. To address this limitation, in this study we aimed to develop a novel oncolytic adenovirus for enhanced infectivity and therapeutic efficacy toward glioblastoma. We developed a novel genetically modified oncolytic adenovirus vector with dual capsid modifications to facilitate infection and specific cytotoxicity toward glioma cells. Modification of the adenoviral capsid proteins involved the incorporation of a synthetic leucine zipper-like dimerization domain into the capsid protein IX (pIX) of human adenovirus serotype 5 (Ad5) and the exchange of the fiber knob from Ad37. The virus infection mechanism and anti-tumor efficacy of modified vectors were evaluated in both *in vitro* (cell) and *in vivo* (mouse) models. Ad37-knob exchange efficiently promoted the virus infection and replication-induced glioma cell lysis by oncolytic Ad5. We also found that gene therapy mediated by the dual-modified oncolytic Ad5 vector coupled with the tumor necrosis factor-related apoptosis-inducing ligand (TRAIL) exhibited significantly enhanced anti-tumor efficacy *in vitro* and *in vivo*. This genetically modified oncolytic adenovirus provides a promising vector for future use in glioblastoma gene-viral-based therapies.

## Introduction

Glioblastoma, the most frequently occurring brain tumor in humans, exhibits a median patient survival time of only approximately 15 months, despite decades of advances in multimodal therapies 1, 2. Recently, oncolytic virotherapy has shown promising efficacy in preclinical and clinical trials for glioblastoma 3, 4. Oncolytic adenovirus based on adenovirus serotype 5 (Ad5), also known as conditionally replicating Ad5 (CRAd5), constitutes a popular alternative approach commonly deployed for cancer therapeutic studies 5. Ad5 infection of cells is mediated by initial anchorage of the globular knob domain of the capsid fiber protein to the cell surface coxsackievirus and adenovirus receptor (CAR) 6, 7. However, CAR is generally expressed at low levels on tumor cells 8, which limits the infectivity and oncolytic efficiency of CRAd5. As the fiber knob constitutes the primary mediator of Ad5 binding to the cell surface, the infectivity of CRAd5 vectors may be improved by genetic modification of this domain. In addition, considering that tropism of infection 9 often differed between adenovirus subgroups, another main approach to modify or broaden the tropism of Ad5-based vectors is fiber knob serotype switching. Numerous Ad5-based oncolytic vectors with chimeric fibers have been developed, with the most advanced with regard to development as preclinical and clinical cancer therapies being Ad5/3 10-12 and Ad5/35 13, 14 resulting from exchange of the Ad3- and Ad35-fiber/knob, respectively.

In comparison, rather than CAR (Ad5-recognized), desmoglein 2 (DSG2, Ad3-recognized), or CD46 (Ad35-recognized), Ad37, belonging to subgroup D, is reported to infect cells by binding sialic acid (SA) 15-18. Ganglioside and membrane glycoproteins containing SA are vital for neurodevelopment and associated with glioma progression 19, 20. Therefore, we hypothesized that genomic modification of CRAd5 via Ad37-knob exchange could modify the tropism of infection and further enhance the infectivity and oncolytic efficacy toward glioblastoma cells.

In addition, protein IX (pIX) exposed on the adenoviral capsid also represents an ideal site for insertion of a tumor-targeting ligand at its C terminus 21, 22. In our previous study, we constructed a novel tumor necrosis factor-related apoptosis-inducing ligand (TRAIL)-modified oncolytic adenovirus vector, termed A4 23. The engineered A4 virus carried the *TRAIL* gene in its genome and presented the TRAIL protein on the viral surface via a Leu-zipper linkage to pIX 23. TRAIL, a typical member of the TNF superfamily, has been demonstrated in numerous studies to specifically induce cancer cell apoptosis by binding to its receptor, TRAILR1 (also known as DR4) or TRAILR2 (also known as DR5), which contain intracellular death domains 24, 25. Moreover, studies have indicated that TRAIL was able to target death receptors on the cancer cell surface 26. Accordingly, the TRAIL modification mediated improved infectivity, tumor-targeting ability, and anti-tumor efficacy of A4 as confirmed in a mouse model bearing human breast cancer 23.

In the present study, we therefore assessed the ability of Ad37-knob exchange of CRAd5 to modify the tropism of infection and enhance virus infectivity and oncolytic efficacy toward glioblastoma cells, and determined whether application of the A4 virus could be broadened to facilitate glioblastoma therapy. Finally, we incorporated TRAIL-modifications on pIX in combination with Ad37-knob exchange to create a novel dual-modified oncolytic adenovirus. We investigated the biochemical characteristics, infectious tropism, and anti-tumor efficiency of the dual-modified adenovirus *in vitro* and *in vivo* to demonstrate its potential utility for glioblastoma therapy.

## Materials and Methods

### Cell lines and cell culture

Human cell lines HEK 293 (embryonic kidney) and U251 (glioblastoma) were purchased from American Type Culture Collection (Bethesda, MD) and cultured in Dulbecco's modified Eagle medium. The QSGC-7701 (human hepatocyte) and U87 (glioblastoma) cell lines were purchased from the Cell Bank of the Type Culture Collection of the Chinese Academy of Sciences (Shanghai, China) and cultured in RPMI 1640 medium and Dulbecco's modified Eagle medium respectively. All cell lines used in this study were authenticated using short tandem repeat profiling within six months of when this project was initiated, and the cells were cultured for less than 1.5 months.

### Analysis of receptors

The mRNA expression of TRAILR1 and TRAILR2 was analyzed using The Cancer Genome Atlas (TCGA) dataset acquired using Firebrowse (http://firebrowse.org). TRAILR1 and TRAILR2 on the surface of cultured cells were stained using PE-labeled anti-human TRAILR1 (Biolegend) and APC-anti-human TRAILR2 (Biolegend). The α2→3 linked SA on cells was recognized by incubation with biotinylated MALII (Vector Laboratories) and subsequent staining with Dylight^R^488 streptavidin (Vector Laboratories). Data of receptor expression were collected via two-color flow cytometry (Becton Dickinson) and analyzed using Cell Quest or Flowjo 7.6.1 software.

### Adenovirus vectors

Adenoviral vectors were generated using AdMax™ Adenovirus Vector Creation Kits (MICROBIX). The adenoviral genomic plasmid pAd5max-zipper, carrying the linker (G4S)3-zipper (R-R34) in the C-terminal end of pIX, was constructed previously 23. Based on pAd5max-zipper, the Ad37-knob gene was further exchanged in the plasmid as illustrated in Figure S1; the resulting plasmid was termed pAd5max-zipper-K37. pAd5max-zipper and pAd5max-zipper-K37 were each co-transfected with the red fluorescence protein (RFP)-containing shuttle plasmid pDC311-RFP-SΔ24E1 into HEK 293 cells for homologous recombination and packaging of the two conditionally replicating adenoviruses CRAd5 and CRAd5/K37. Conditional replicating adenoviruses A3 and A4 were packaged in our previous study 23; A4/K37 was generated by co-transfection with pAd5max-zipper-K37 and pDC311-zTRAIL-RFP-SΔ24E1 in HEK 293 cells. All virions were purified using CsCl equilibrium density gradient centrifugation 27; the titers were determined by the 260-nm absorbance method 28.

### Virus infection analysis

Virus-mediated reporter gene (*RFP*) expression can be used to reflect virus infectivity toward cells. In brief, cells were incubated with adenoviral vectors at the same multiplicity of infection (MOI; virus particles (VPs) per cell) for 16 h, then RFP expression was imaged by fluorescence microscopy (Nikon) and quantified by fluorescence-activated cell sorting (FACS).

To further analyze the infectivity of viruses, soluble trimeric Ad5-knob and Ad37-knob were used to block receptors. Cells were incubated with Ad5-knob or Ad37-knob at 4°C for 30 min then viruses were added for subsequent incubation at 4°C for 1 h. After being washed twice with PBS, cells were harvested for virus quantification by quantitative PCR (qPCR) or virus-infected cells were analyzed by FACS after being cultured at 37°C for 16 h.

For cell binding analysis, cells were incubated with viruses at 4°C for 1 hour and washed twice with ice-cold PBS, then fixed immediately for subsequent immunostaining. For internalization analysis, cells were first incubated with viruses at 4°C for 1 h with subsequent incubation at 37°C, then fixed and immunostained after being washed twice with PBS. Rabbit anti-adenovirus (Abcam, 1:1000) and CoraLite488 conjugated goat anti-rabbit IgG (Proteintech, 1:500) were employed for detection of adenovirus in cells. The images were taken by laser scanning confocal microscope (Zeiss) and quantified using image-pro-plus-6.0 software.

### Cell viability assay

Adenovirus-induced cell death was assessed using the MTT assay. Cells were infected with adenoviruses at various MOIs (0-10,000 VPs per cell) and the cell viability was measured after 72 h. The cell viability ratio (%) was calculated according to the following formula: [(experimental group absorbance - background absorbance) / (control group absorbance - background absorbance)] × 100%.

### Biodistribution analysis of adenoviruses in Balb/c mice

Female Balb/c mice (18-20 g, Liaoning Changsheng Biotechnology) bearing no tumors were used to analyze the biodistribution of Ad37-knob-exchanged adenoviral vectors. Bio-photonic imaging of virus-mediated RFP expression was detected using an Fx Pro Imaging System (Kodak) 29 at 72 h post virus injection (1 × 10^11^ VPs per mouse, intravenously (i.v.)).

Viral genomes in tissues were quantified by quantitative real time-PCR at 72 h post virus administration (1 × 10^11^ VPs per mouse, i.v.). DNA was extracted from tissues using the MiniBEST Universal Genomic DNA Extraction Kit (TaKaRa) and quantified using a Nanodrop spectrophotometer (Thermo Scientific). Then, 50 ng of DNA containing viral genomes was quantified using SYBR green (TaKaRa) real-time PCR (Applied Biosystems) using hexon primers (sense: 5′-TGGGCATCCTACACCAACAC-3′; anti-sense: 5′-AGTGCGCCCATGGACATAAA-3′).

### Dot blot analysis

VPs (1 × 10^11^) were exposed to a nitrocellulose membrane using a dot blot chamber. The membrane was sequentially incubated with blocking reagent (3% skimmed milk in TBS), primary antibodies [anti-TRAIL (1:200, Developmental Studies Hybridoma Bank), rabbit anti-adenovirus (1:3000, Abcam), anti-K5 (1:500, rabbit serum prepared after three times of immunization), or anti-K37 (1:500, rabbit serum prepared after three times of immunization)] and horseradish peroxidase (HRP)-labeled secondary antibodies [HRP-labeled rabbit anti-mouse IgG (1:5000, Jackson Immuno-Research), or HRP-labeled goat anti-rabbit IgG (1:5000, Jackson Immuno-Research)], and finally developed with diaminobenzidine. The membrane was washed with TBST (TBS with 0.2% Tween-20) three times after every incubation.

### Apoptosis analysis

Cells plated in 12-well plates were infected with viruses at 1000 MOIs (VPs per cell). At 24-hours post-infection, cells were harvested and stained using FITC-labeled annexin V antibodies (Beckman Coulter). The annexin V positive cells were analyzed by flow cytometry.

### Therapeutic experiments in xenograft tumor models

Female Balb/c nude mice (Vital River Laboratories) at 6-8 weeks old were used in this study. Freshly cultured U87 cells (1 × 10^6^) were injected subcutaneously into the left flank of mice. Oncolytic adenoviruses were administrated (i.v.) when the tumor volume reached approximately 100 mm^3^. Mice in groups were treated with PBS or adenoviral vectors at a total of 3 × 10^10^ VPs in three consecutive days. Tumor size was measured every two days and the tumor volume was calculated as (length × width^2^)/2. At the end of the experiment, mice were sacrificed under anesthesia. Tumors were dissected for imaging, weight, and other analyses.

All animal procedures were conducted in strict accordance with the National Institutes of Health Guide for the Care and Use of Laboratory Animals and were approved by the University Committee on the Use and Care of Animals of Jilin University of China (approval NO. SY0306).

### Immunohistochemistry

Tumor sections (5-μm thick) were immunostained to quantify adenovirus and cleaved caspase 3. In brief, appropriate primary antibodies [goat anti-cleaved caspase 3 (diluted 1:200, Cell Signaling Technologies), rabbit anti-adenovirus (diluted 1:3000, Abcam), or rabbit anti-Ki67 (diluted 1:200, Merck Millipore)] were incubated with sections at 4ºC overnight. The HRP-labeled secondary antibodies [goat anti-mouse IgG/HRP (ZSGB-BIO) or goat anti-rabbit IgG/HRP (ZSGB-BIO)] for immunochemical staining were developed via a peroxidase reaction with diaminobenzidine as the chromogen.

### Statistical analysis

An unpaired t-test or one-way analysis of variance (ANOVA) followed by the Newman-Keuls test was performed to analyze the data. The results are expressed as the means ± S.E.M. and are considered significant at *P* < 0.05.

## Results

### Ad37-knob exchange improves the infectivity of CRAd5 toward glioblastoma cells

**Figure 1:**
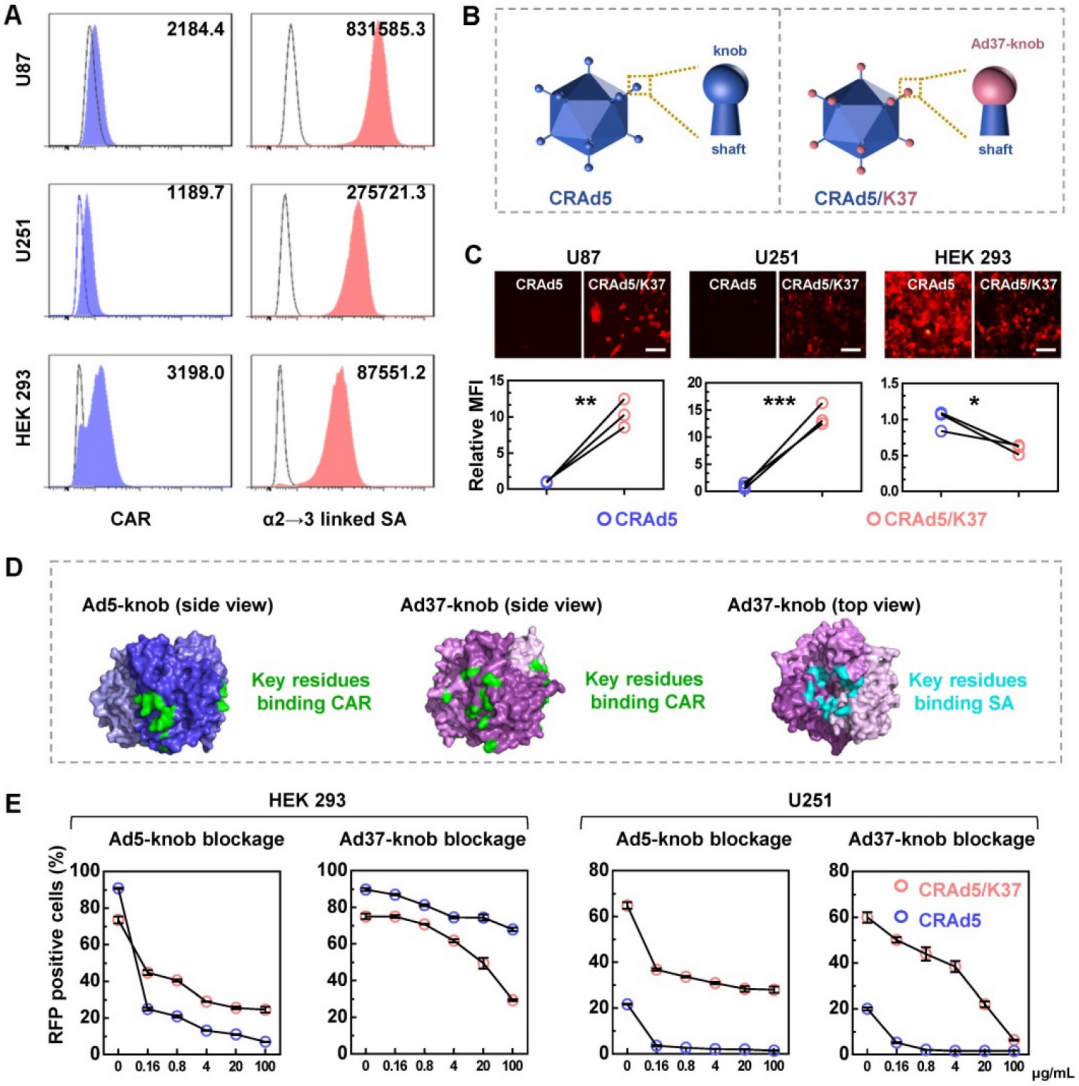
Broadened infection mechanism mediated by Ad37-knob replacement. (**A**) The expression of CAR and α2→3 linked SA on glioma cells was analyzed by FACS. (**B**) Differences between the CRAd5 and CRAd5/K37 capsid. (**C**) Virus-mediated RFP expression was detected at 16 h post infection at the same MOIs (1000 VPs per cell). The relative MFI of RFP expression was analyzed from the MFI values detected by FACS. Scale bar = 100 µm. (**D**) The key residues of the CAR binding site on the trimeric Ad5-knob (PDBID: 6hcn) and Ad37-knob (PDBID: 4keu), along with key residues of the SA binding site on the Ad37-knob are summarized. (**E**) Cells preincubated with soluble trimeric Ad5-knob protein or Ad37-knob protein were infected by viruses and the RFP positive cells were counted by FACS at 24 h post infection. K5, soluble Ad5-knob; K37, soluble Ad37-knob. Error bars were calculated from at least three values. Significant differences were analyzed using an unpaired T test between two groups; *P < 0.05, **P < 0.01, ***P < 0.001.

We assumed that Ad37-knob replacement would allow the modified adenovirus to recognize SA as a receptor. To determine the suitability of this strategy for enhancing adenoviral infectivity toward glioma cells, we measured the expression of CAR and SA that can be recognized by Ad37 in human glioma cell lines (Figure 1A). CAR was expressed at a low-level on the surface of U87 and U251 cells, which also exhibited a low CAR-positive percentage, whereas the α2→3 linked SA was more abundantly distributed compared with that on HEK 293 cells.

Next, we designed an Ad37-knob-exchanged oncolytic adenovirus CRAd5/K37 (Figure 1B, Figure S2). The viral infectivity, reflected by virus-mediated reporter gene (RFP) expression (Figure 1C), showed that Ad37-knob-exchanged adenovirus-mediated RFP expression exhibited an 8- to 16-fold increase in glioma cells and a significant decrease in HEK 293 cells, compared with that of CRAd5.

It has been reported that both SA and CAR binding sites coexist on the Ad37 fiber knob, even though Ad37 uses SA rather than CAR to infect cells 17, 30. A summary of the binding sites indicated from structural studies on the Ad5-knob 31, 32 and Ad37-knob 17, 33 is shown in Figure 1D. In addition, Wu *et al.* found that the relatively short, inflexible Ad37 fiber shaft restricted interactions with CAR at the cell surface, whereas Ad5 with Ad37-knob change and the remaining Ad5-shaft were able to use CAR for internalization 34. Thus, we explored the types of receptors used by CRAd5/K37 for infecting cells. Soluble trimeric Ad5-knob or Ad37-knob protein was used to block receptors during adenovirus infection. As shown by both virus-mediated RFP expression (Figure 1E) and virus binding (Figure S3), CRAd5/K37-mediated infection was inhibited by Ad5-knob blockage in HEK 293 cells and U251 cells, indicating that CAR also served as a receptor of the Ad37-knob-replaced adenovirus. In HEK 293 cells, soluble Ad37-knob blockage inhibited infection of CRAd5/K37 more efficiently than that of CRAd5. In U251 cells, CRAd5/K37-mediated RFP positively expressing cells could be decreased to 6.3% by excess soluble Ad37-knob blockage (100 μg/mL), compared with 26.8% as reduced by soluble Ad5-knob (100 μg/mL). Collectively, we concluded that Ad37-knob replacement improved the infectivity of Ad5 for glioma cells via a broadened infection tropism dependent on SA as well as CAR.

### Oncolytic efficiency of CRAd5/K37 *in vitro* and *in vivo*

**Figure 2:**
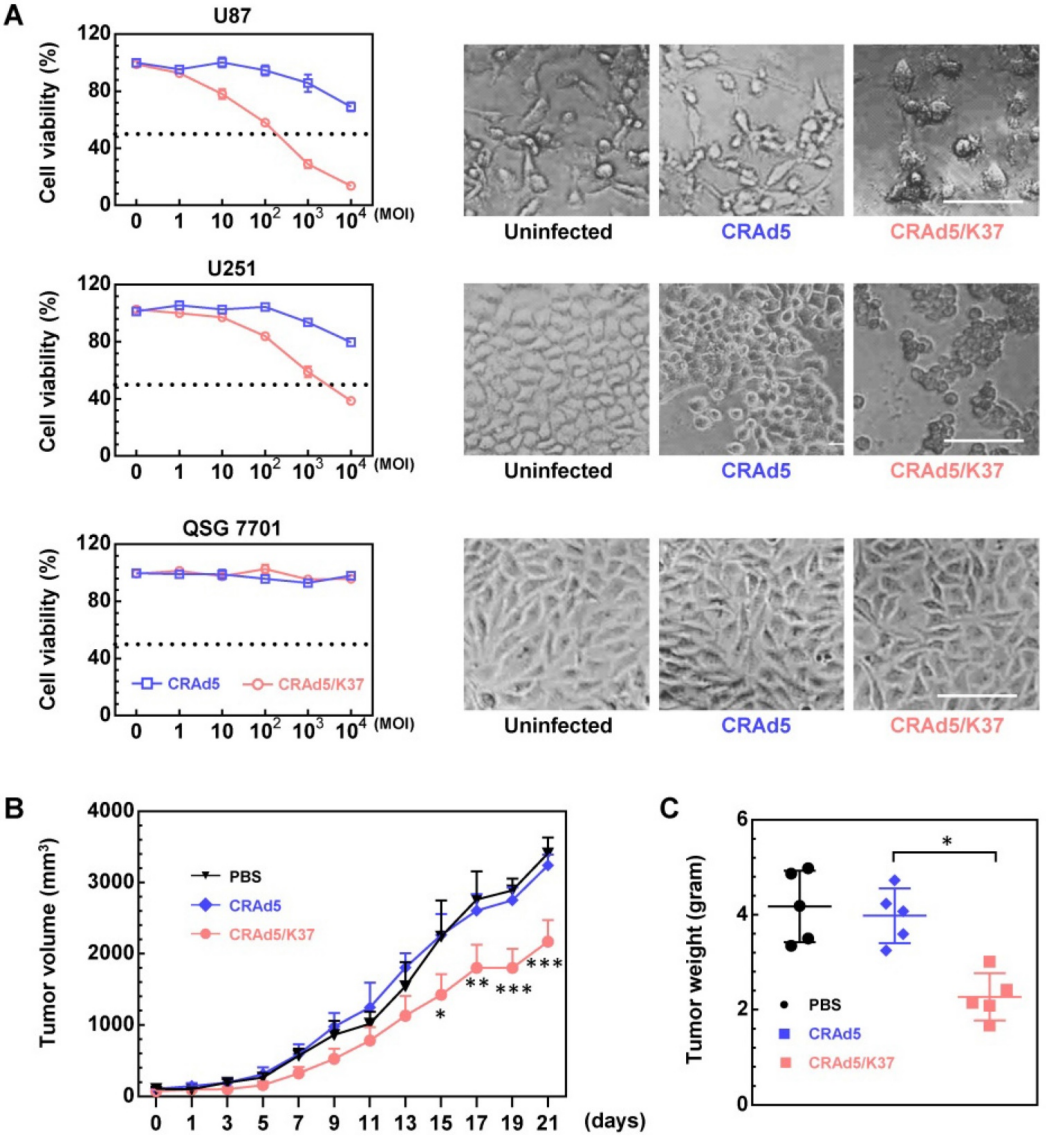
Oncolytic effects of viruses *in vitro* and *in vivo*. (**A**) Cell death mediated by oncolytic adenoviruses was analyzed by MTT assays at 72 h post virus infection, and the morphology of cells uninfected or infected by viruses (MOI = 10,000) was recorded. Scale bar = 100 µm. Significant differences were analyzed by one-way ANOVA among groups. ***P < 0.001. (**B**) Oncolytic effect of CRAd5 and CRAd5/K37 was evaluated in U87-tumor bearing nude Balb/c mice; when tumors reached approximately 100 mm^3^, viruses were administered (intratumor). PBS or total 3 × 10^10^ VPs of viruses were injected into mice for three consecutive days (day 1 to day 3); tumor volume was recorded every 2 days. Each group contained at least six mice. Indicated significance is between CRAd5/K37 and CRAd5 groups. (**C**) Tumor weight was analyzed at the end of the *in vivo* experiment. Significant differences were analyzed using one-way ANOVA among groups; *P < 0.05, **P < 0.01, ***P < 0.001.

The fiber knob-modified adenovirus was able to better dissolve glioma cells compared to the unmodified adenovirus; moreover, both adenoviral vectors showed no cytotoxicity in normal liver cells, as demonstrated by MTT assay along with cell morphology (Figure 2A). The superiority of Ad37-knob-exchanged adenovirus with regard to oncolytic effect was further confirmed in U87 tumor-bearing Balb/c nude mice. From the results of tumor volume (Figure 2B) and tumor weight (Figure 2C), tumor growth was significantly inhibited in mice administrated with CRAd5/K37, whereas the unmodified CRAd5 exerted no significant therapeutic effects *in vivo*.

### Biodistribution of CRAd5/K37 in Balb/c mice

**Figure 3:**
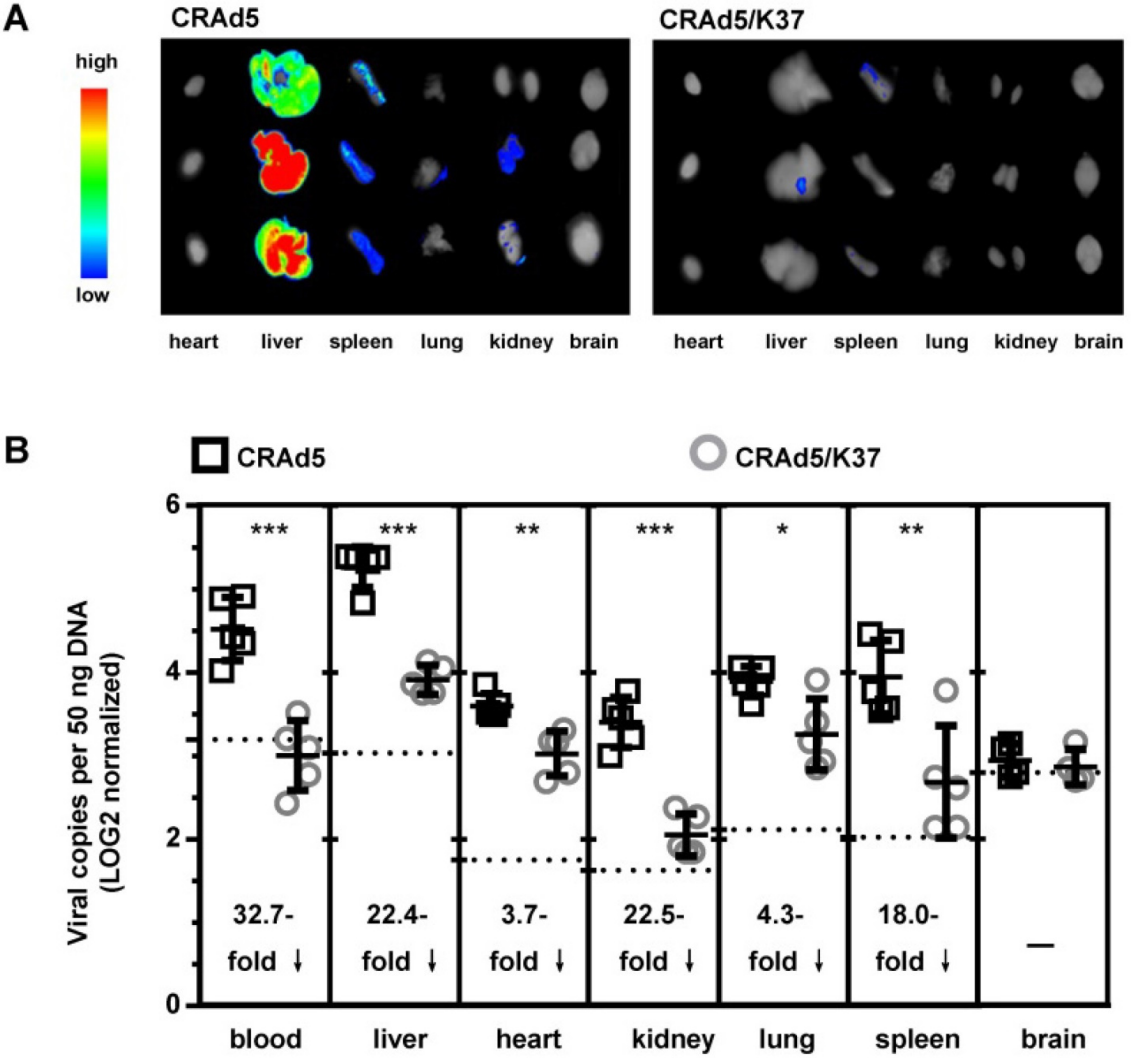
Biodistribution of adenoviruses. (**A**) Imaging of adenovirus-mediated RFP expression in organs at 72 h post virus injection (i.v.). (**B**) Viral load in the blood and organs was measured by qPCR at 72 h post virus injection; background signals from control injections with PBS are indicated by dashed lines. A total of five mice were included in each group. *P < 0.05, ***P < 0.01, ***P < 0.001.

To further assess the *in vivo* tropism of virus infection, Balb/c mice were injected i.v. with CRAd5 and CRAd5/K37. Virus mediated RFP expression was imaged and viral load was quantified by qPCR at 72 h post virus injection (Figure 3). As expected, and previously documented 35, capsid-unmodified Ad5 vectors were mainly localized in the liver and spleen. However, CRAd5/K37 virus-mediated RFP expression was weakly detected in all six examined organs at 72 h post injection. This indicated that the Ad37-knob replacement of Ad5 decreased infectivity for normal tissues and cells in Balb/c mice.

### Construction of the dual-modified oncolytic adenoviral vector

**Figure 4:**
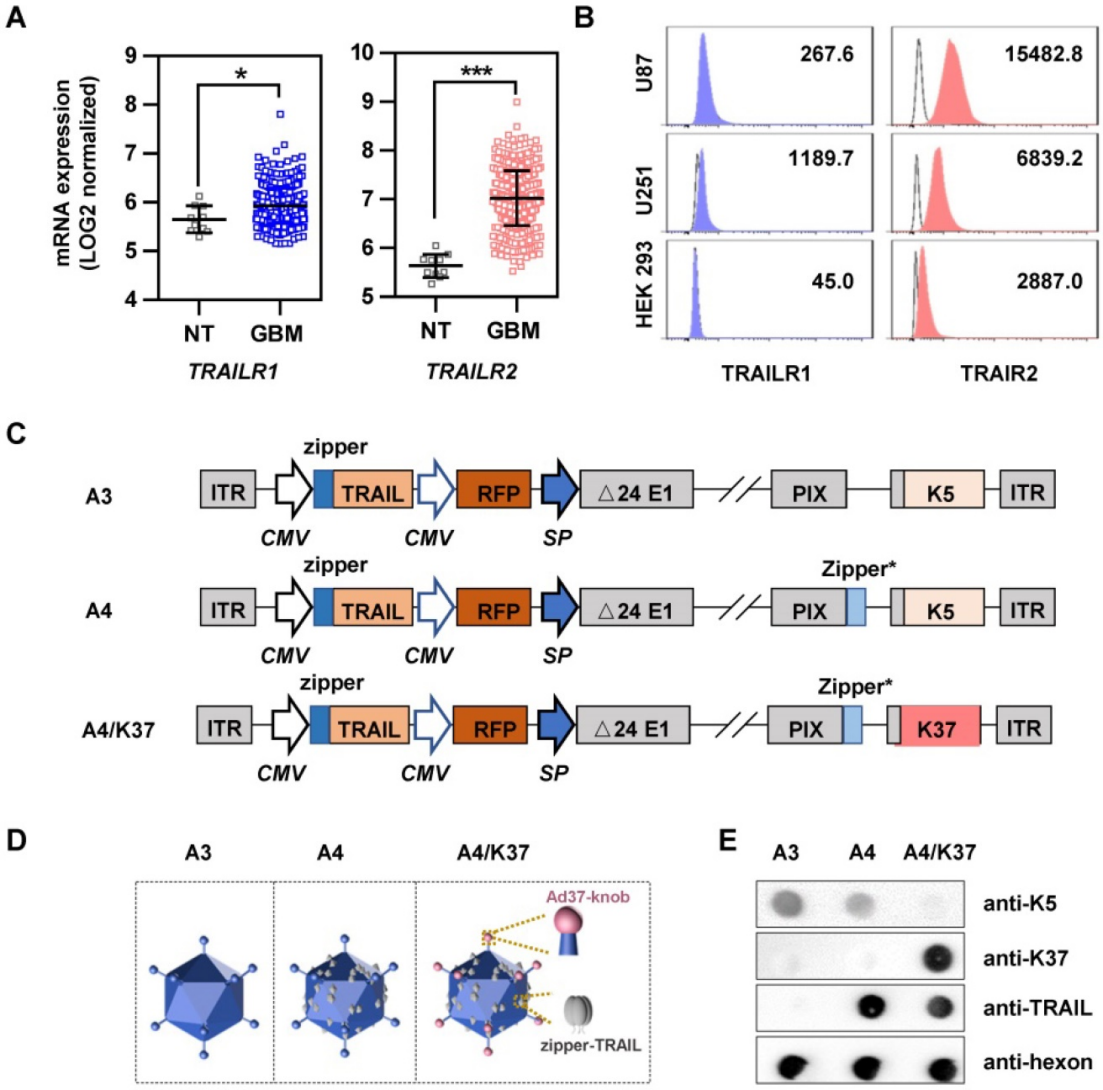
Analysis of TRAIL-related receptors in glioblastoma and construction of the dual-modified oncolytic adenovirus vector. (**A**) mRNA expression of TRAILR1 and TRAILR2 in normal tissue (NT, *n* = 10) and glioblastoma (GBM, *n* = 431) was analyzed using the TCGA dataset. (**B**) The expression of TRAIL-related death receptors was measured by FACS using fluorescent dye-labeled antibodies. (**C**) Genomic design of oncolytic adenoviruses; K5, Ad5-knob; K37, Ad37-knob. (**D**) Differences of viral capsid modifications. (**E**) Determination of capsid modifications by dot blot. *P < 0.05, **P < 0.01, ***P < 0.001.

In addition to fiber switching, TRAIL modification on capsid protein IX via a Leu-zipper linkage also serves as a good strategy to improve the selective and efficient infection of cancer cells 23. To determine whether the TRAIL-modification strategy was suitable to facilitate targeted therapy of oncolytic adenovirus for glioblastoma, we first analyzed the mRNA expression of TRAIL-related death receptors using the TCGA dataset (Figure 4A). Compared with that in normal tissues, TRAILR1 and TRAILR2 were both significantly more highly expressed in glioblastoma tissues. In addition, TRAILR1 and TRAILR2 were highly expressed on the surface of U87 and U251 cells, especially the latter (Figure 4B). We also analyzed the sensitivity of glioma cells to TRAIL. TRAIL protein incubation resulted in U87 and U251 cell death by inducing apoptosis, as shown by MTT assay (Figure S4A) and annexin V-propidium iodide double staining assay (Figure S4B). Therefore, A4 virus carrying *TRAIL* as a therapeutic gene and modified with TRAIL on the capsid protein pIX may be suitable and selective for glioblastoma treatment.

We next applied Ad37-knob exchange and TRAIL modification to adenovirus, finally obtaining a dual-modified oncolytic adenovirus, termed A4/K37. By rebuilding the genomes (Figure 4C), adenoviruses with/without modifications on the capsid were generated; capsid differences are shown in Figure 4D. In brief, the capsid of A3 virus was not modified, A4 virus was equipped with TRAIL on capsid protein pIX, and the A4/K37 virus was dual-modified with Ad37-knob exchange and TRAIL linkage on the capsid.

The capsid modifications on adenoviral vectors were analyzed by dot-blot assay (Figure 4E). As expected, Ad5-knob could be detected on A3 and A4, but was undetected on A4/K37; conversely, Ad37-knob was only stained on A4/K37. Immunoreaction for TRAIL on the surface of A4 and A4/K37 was positive in each case.

### Analysis of the infection and targeting ability of the dual-modified adenovirus vector

**Figure 5:**
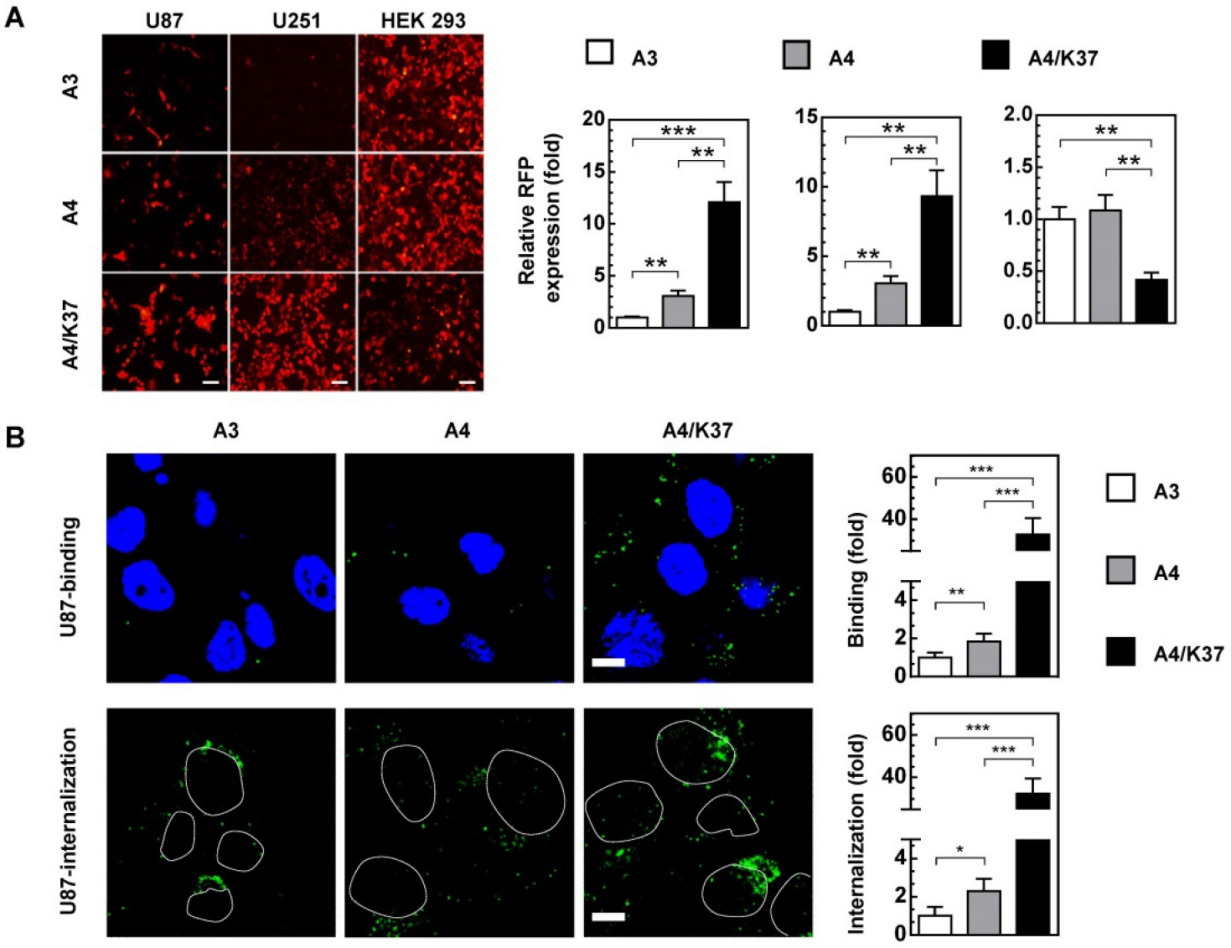
Infection and targeting ability of viruses. (**A**) Virus-mediated RFP expression. Cells were incubated with viruses (1000 MOI), and virus-mediated RFP expression was detected by fluorescence microscopy and quantified by FACS at 16 h post infection. Error bars are from values of three independent repeated experiments**.** Scale bar = 100 µm. (**B**) Binding and internalization during the virus infection mechanism in U87 cells was analyzed via immunostaining for adenovirus. Images show representative confocal stacks. Nuclei (DAPI stain) are shown in blue and virus particles are labeled green in binding analysis; nuclei are shown as outlines in the images of internalization. Scale bar = 10 µm. *P < 0.05, **P < 0.01, ***P < 0.001.

Based on our hypothesis, each modification would enhance the infectivity of adenovirus to glioma cells. In glioma cells, single modification with TRAIL showed an approximately 2 to 3.5-fold enhancement of virus-mediated RFP expression, whereas the dual-modification-induced increase was more than 10-fold compared with that of the A3 virus. In HEK 293 cells, TRAIL on the viral surface did not influence the infectivity, whereas this was reduced by Ad37-knob exchange (Figure 5A).

To further clarify which steps of infection were influenced by the dual-modifications, virus binding and internalization was analyzed (Figure 5B, Figure S5). The cell binding ability of adenovirus was enhanced 1.5 to 2.5-fold by TRAIL modification, and further improved more than 30-fold in glioma cells by Ad37-knob replacement. The internalization of adenoviral vectors showed similar fold-changes. In comparison, the cell binding and internalization were not altered by TRAIL modification, although cell binding was weakened by Ad37-knob exchange in HEK 293 cells. Together, the dual modifications altered viral infectivity by influencing the cell binding process, whereas the modifications appeared to have minimal influence on virus internalization.

### Cytotoxicity analysis of dual-modified oncolytic Ad5 vectors *in vitro*

**Figure 6:**
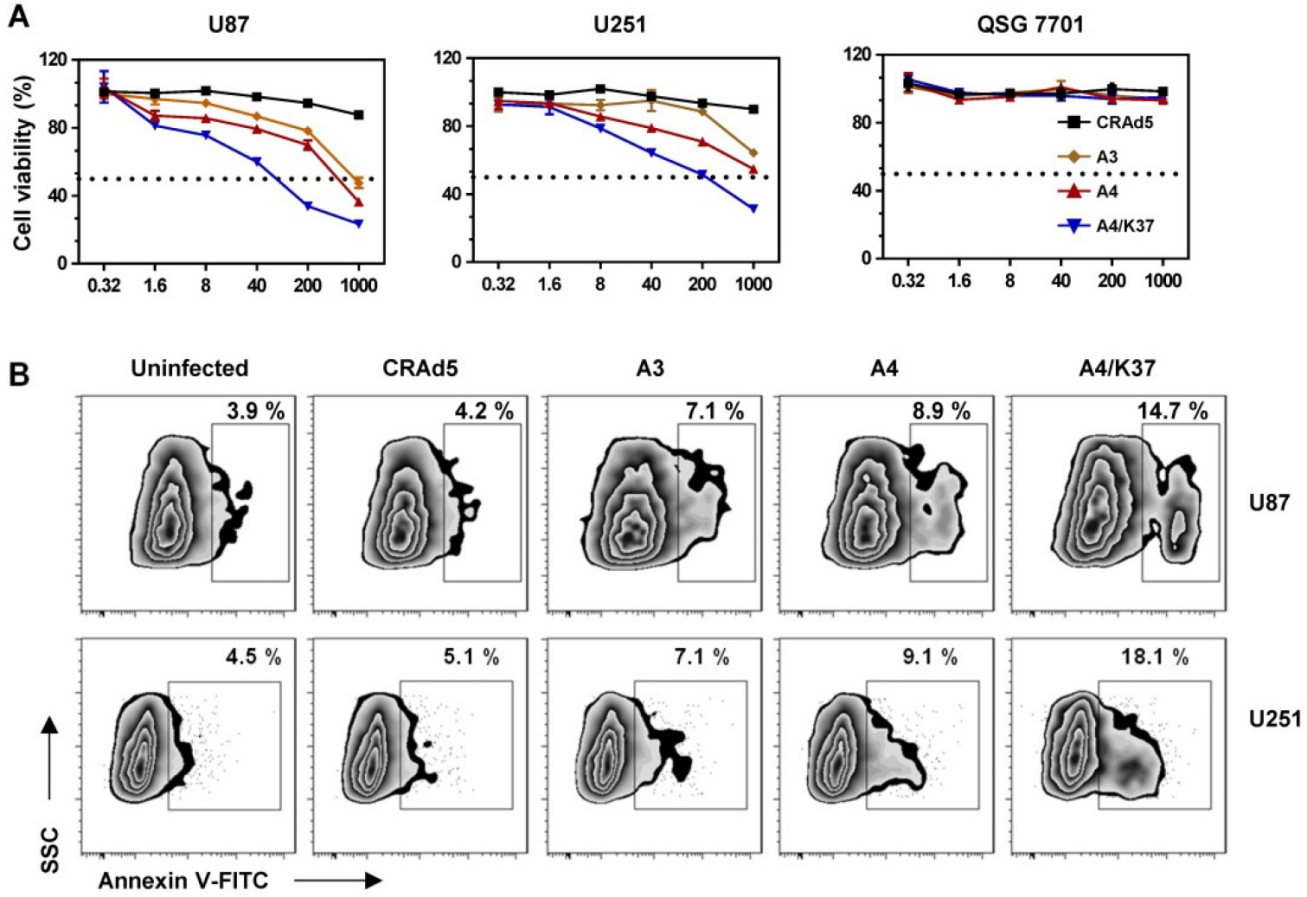
Anti-tumor efficacy of TRAIL-carrying oncolytic adenoviruses *in vitro.* (**A**) Cell viability was measured by MTT assay at 72 h post virus infection. Significant differences were analyzed by one-way ANOVA among groups. ***P < 0.001. (**B**) Annexin V was stained and analyzed by FACS at 24 h post virus infection (1000 MOI).

In order to check the cancer-specific replication of viruses and the advantages of dual-modifications for anti-tumor activity, the toxicity of viruses was evaluated in glioma cell lines and a normal liver cell line following long-term (72-h) incubation with oncolytic viruses (Figure 6A). In glioma cells (U87, U251), incubation of viruses at different MOIs revealed their ability to inhibit cell growth. Specifically, A3 virus carrying the *TRAIL* gene more effectively inhibited cell growth compared to CRAd5 with no therapeutic genes, indicating that *TRAIL*-mediated gene therapy is effective in glioma cells; TRAIL modification on pIX also improved the anti-tumor efficiency (A4 *vs.* A3). Dual modifications exerted further improved tumor growth inhibition, compared with that of single TRAIL modification (A4/K37 *vs.* A4). In contrast, viruses exhibited no cytotoxicity to normal liver cells.

Annexin V expression of glioma cells was analyzed following relative short (24-h) period of virus incubation (Figure 6B). The oncolytic effects of unmodified CRAd5 did not result in obviously increased expression of annexin V (CRAd5 *vs.* uninfected), whereas inclusion of the *TRAIL* gene accelerated the apoptosis of glioma cells (A3 *vs.* CRAd5). In turn, TRAIL modification on the viral surface further promoted cell apoptosis (A4 *vs.* A3), with the dual-modified adenovirus-mediated *TRAIL* gene therapy being able to efficiently induce glioma cells apoptosis (A4/K37 *vs.* other groups).

### Anti-tumor activity of oncolytic adenoviruses in a U87 xenograft tumor model *in vivo*

**Figure 7:**
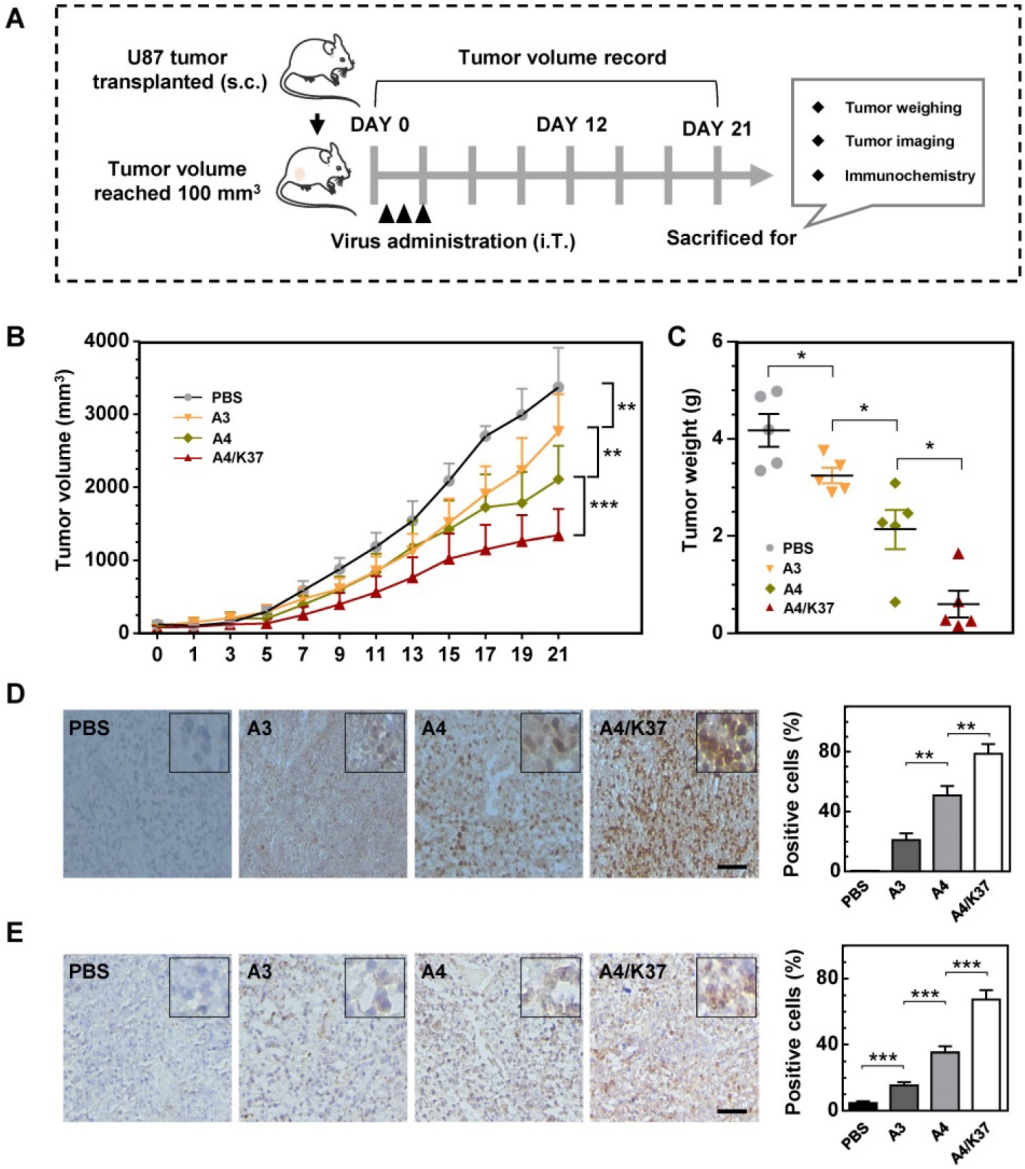
Anti-tumor efficacy *in vivo.* (**A**) Animal experimental procedures; s.c., subcutaneous; i.T., intra-tumor. (**B**) Tumor growth curve; each group included at least six mice; (**C**) Tumor weight; (**D**) immunochemical analysis for adenovirus distribution in tumors; positive cells were quantified. Scale bar = 50 µm. (**E**) Immunochemical staining for cleaved caspase-3 in tumors; positive cells were quantified. Scale bar = 50 µm. Significant differences were analyzed by one-way ANOVA among groups; *P < 0.05, **P < 0.01, ***P < 0.001.

The anti-tumor activity of the dual-modified adenovirus vector was next evaluated in tumor-bearing mice via intratumoral administration (Figure 7A). The results of tumor growth kinetics (Figure 7B) showed that all adenovirus vectors inhibited tumor growth compared to that of the PBS-treated group; the tumor inhibition was enhanced by TRAIL display on the viral capsid surface (A4 *vs.* A3) and further improved by dual modifications (A4/K37* vs.* A4). At the end of the experiment, mice were killed and tumors were taken for imaging and weighing. The results of both morphological size (Figure S6) from images and tumor weight (Figure 7C) also supported the conclusion that the dual-modified A4/K37 virus exhibited the best anti-tumor activity.

Immunochemical analysis of adenoviral vectors in the tumor (Figure 7D) revealed that adenovirus was not detected in the PBS group, whereas the adenovirus positive cells increased in the sequence: A3 < A4 < A4/K37. A similar trend was shown by cleaved caspase 3 analysis (Figure 7E). Therefore, we concluded that dual-modification promoted virus replication in the tumor and that virus-mediated *TRAIL* gene therapy ultimately optimized the tumor inhibition efficacy *in vivo.*

## Discussion

Currently, oncolytic adenoviruses developed based on fiber-modified Ad5 vectors afford improved effects in cancer therapies in clinical trials, as represented by Ad-RGD 36, 37, Ad5/3 10-12, and Ad5/35 14. Although recombinant Ad5 vectors with full-length Ad37-fiber 38 or Ad37-knob exchange 34 have been generated for purposes of studying the infection mechanisms, to our knowledge no oncolytic adenovirus-based Ad37-fiber/knob chimeric Ad5 has previously been developed. Here we generated an Ad37-knob-exchanged oncolytic Ad5 (CRAd5/K37) and determined that Ad37-knob exchange mediated increased infectivity toward glioma cells in accordance with their high SA expression (Figure 1A-1C). As SA and CAR binding sites uniquely co-exist on the Ad37-knob, we further confirmed that CRAd5/K37 obtained an extra SA-dependent infection mechanism, whereas the weakened CAR-dependent infectivity was retained (Figure 1D, 1E). This feature differs from that of other fiber/knob chimeric Ad5 vectors that exhibit solely CAR-independent infection of cells. As all GBM tissues do not uniformly over-express the same receptors, CRAd5/K37 may therefore be more conducive to transduction in glioma tissue.

CRAd5 exhibited only slight oncolytic effect *in vitro*, even when the MOI reached as high as 10^4^ VPs/cell (Figure 2A). Only when the MOI was further increased up to 10^5^ VPs/cell could approximately 60% cytotoxicity be observed (data not shown). *In vivo*, CRAd5 showed no therapeutic effects, reflecting the limited infectivity and oncolytic effect of unmodified oncolytic adenovirus toward glioblastoma (Figure 2B, 2C). In contrast, CRAd5/K37 afforded significant inhibition of tumor growth, which was demonstrated via enhanced infectivity toward glioma cells *in vitro*. Notably, all the oncolytic adenoviruses in this study were designed with Δ24E1a 39 under a tumor specific promoter (survivin promoter) 40 to ensure the tumor-specific replication of the viruses, demonstrating no significant toxicity in normal cells *in vitro* (Figure 2A, 6A) and no obvious damage to the liver of virus-treated mice (Figure 2 D, 7F).

The discovery that TRAIL can induce apoptosis of cancer cells without causing toxicity in mice has led to the wide application of TRAIL in cancer therapy studies 13, 41. Combined with the expression analysis of TRAIL-related death receptors via the TCGA dataset and the results of TRAIL-induced apoptosis in glioma cell lines (Figure 4A, 4B, Figure S4), it was concluded that TRAIL may facilitate targeting and apoptosis induction in glioblastoma therapies. Moreover, oncolytic adenovirus infection can result in the upregulation of pro-apoptosis proteins (e.g., BAX, BIM, and BIK) without resulting in the activation of caspases 42-45, whereas TRAIL is able to induce apoptosis via caspases along with the transmission of BAX and BIM 41, 46. Therefore, the modification/arming of oncolytic adenovirus with TRAIL may constitute an ideal strategy to enlist the apoptosis-related mechanisms induced thereby 47-49. Oncolytic adenovirus A4 with TRAIL modified on the C terminus of pIX offered an excellent platform for cancer-targeting gene-viral-therapy. By confirming the superiority of CRAd5/K37 with regard to infectivity and oncolytic effects, we applied Ad37-knob exchange to further optimize the A4 virus for treating glioblastoma. The novel oncolytic adenovirus (A4/K37) contained the *TRAIL* gene in its genome and harbored dual capsid-modifications of Ad37-knob modification and TRAIL protein linkage on pIX (Figure 4C-4E). A4/K37 equipped with Ad37-knob modification based on A4 exhibited optimized infectivity toward glioma cells, along with decreased binding to HEK 293 cells (Figure 5A, 5B, Figure S5), compared to those of the A4 virus. A4/K37 also showed enhanced anti-tumor efficacy compared with that of A3 and A4 viruses *in vitro* (Figure 6) and *in vivo* (Figure 7, Figure S6). Furthermore, compared with other studies of oncolytic adenovirus-based *TRAIL* gene therapy for glioblastoma 13, 50, A4/K37 revealed different capsid characteristics and infection mechanism induced by the dual modifications.

**Figure 8:**
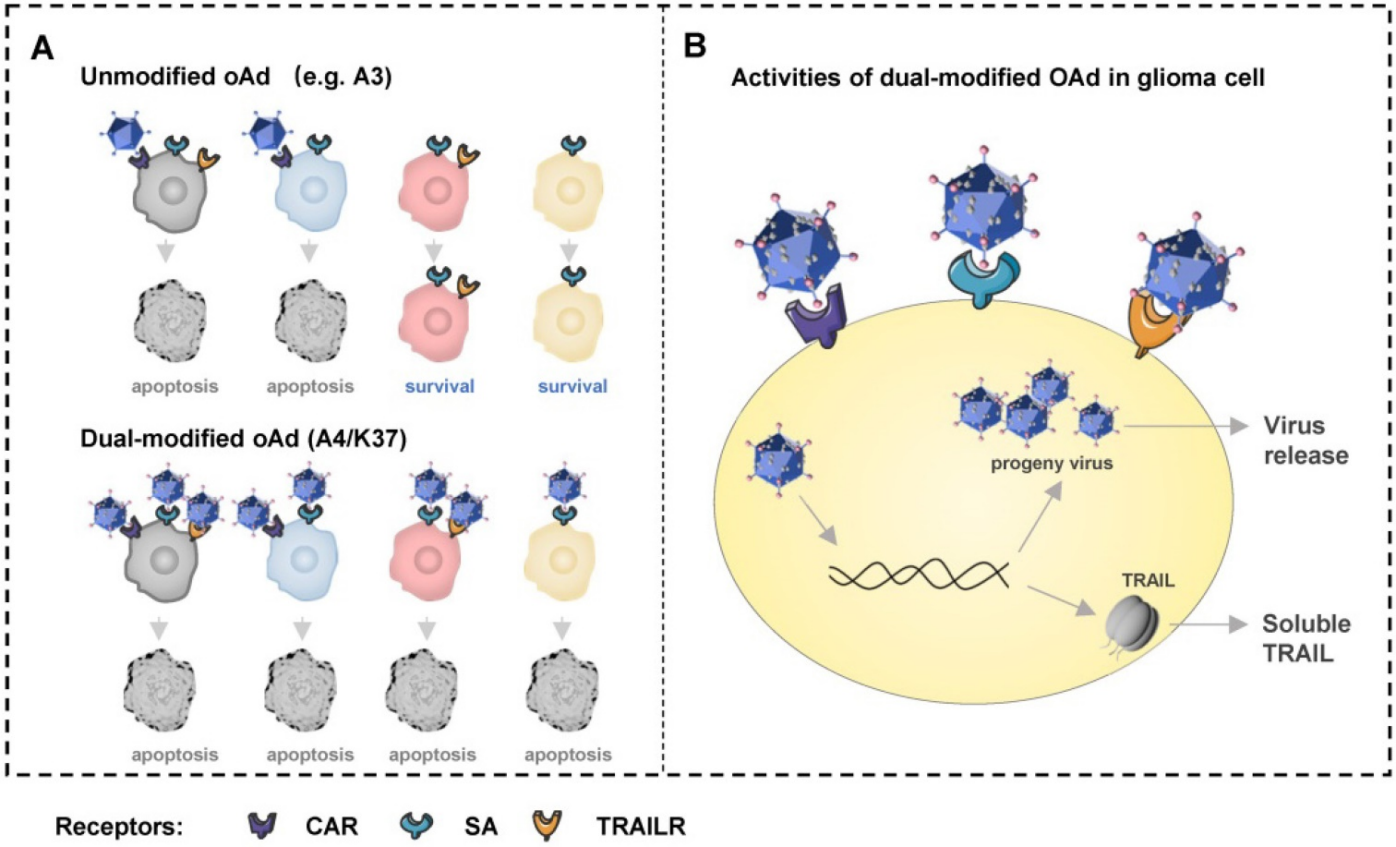
Schematic overview of dual-modified oncolytic adenovirus (OAd) for glioblastoma therapy. (**A**) Unmodified oncolytic adenovirus can only infect and kill glioma cells expressing CAR, but the dual-modified oncolytic adenovirus is able to infect and kill cells with different expression patterns of receptors via three types of receptors. Cells in four colors represent cells expressing three types of receptors (grey), cells expressing CAR and SA (blue), cells expressing SA and TRAILRs (pink), cells only expressing SA (orange). (**B**) The dual-modified oncolytic adenovirus will replicate to generate progeny viruses and mediate TRAIL expression after entering cell. Progeny viruses will be release to infect new glioma cells after cell lysis. Soluble TRAIL secreted can induce glioma cells apoptosis via TRAILR1 or TRAILR2.

In summary, we described an Ad37-knob exchange strategy for developing oncolytic adenovirus vectors and reported a novel dual-modified oncolytic adenovirus, A4/K37, for enhanced GBM therapy (Figure 4C-4D, Figure 8). This virus shares different capsid modifications with the reported oncolytic adenovirus for glioblastoma therapy, and is capable to infect cells via three types of receptors (CAR, SA, and TRAILRs), resulting in the advantages of A4/K37 to enter and kill heterogeneous glioma cells with the non-uniform expression of cell surface receptors, although further studies using patients' glioblastoma tissues *in vitro* and in patient-derived tumor xenograft (PDX) mouse models are warranted (Supplementary Table). Based on this work, more innovative modification approaches might be introduced to further increase the potential use in the treatment of GBM, especially those aiming to promote the i.v. administration of this virus.

## Supplementary Material

Supplementary figures and tables.

## Abbreviations

GBM: glioblastoma multiforme
BBB: blood-brain barrier
TRAIL: tumor necrosis factor-related apoptosis-inducing ligand
Ad: adenovirus
CRAd5: conditionally replicating Ad5
CAR: coxsackie and adenovirus receptors
DSG2: desmoglein 2
SA: sialic acid
pIX: protein IX
TCGA: The Cancer Genome Atlas
hCMEC/D3: immortalized human brain capillary endothelial cell
