# Supplementary material for: A tropism-transformed Oncolytic Adenovirus with Dual Capsid Modifications for enhanced Glioblastoma Therapy
Source: J Cancer. 2020 Jul 29;11(19):5713–26. doi: 10.7150/jca.46463 (PMC7477443; doi:10.7150/jca.46463)
Supplement: Supplementary file 1 — Supplementary figures and tables. [file jcav11p5713s1.pdf]

Supplementary Figures and Legends

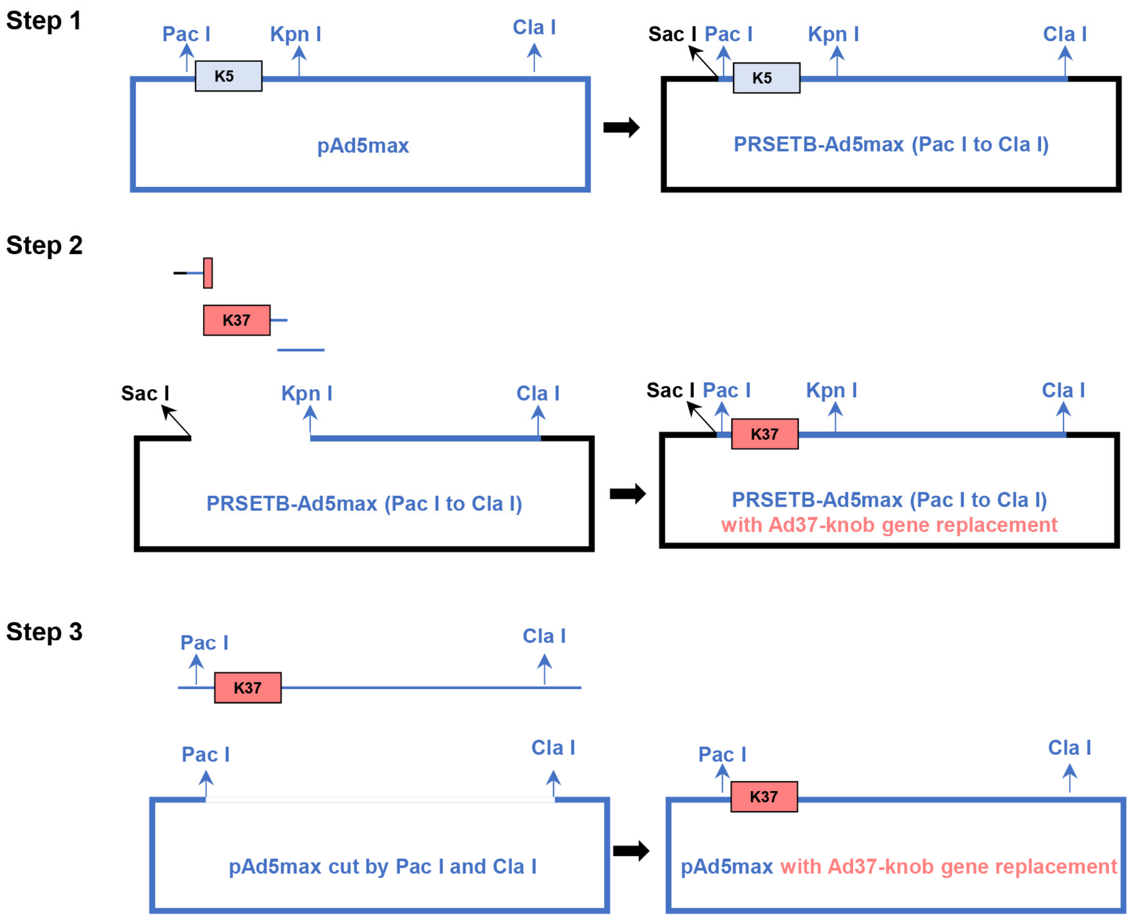

Figure S1

Construction of Ad37-knob gene-exchanged Ad5max plasmid. The final plasmid was obtained through three steps. Firstly, the sequence of Ad5max from the PacI site to ClaI was cloned into a transition PRSETB vector; secondly, the Ad5-knob region was exchanged with the Ad37-knob

gene in the constructed transition vector; and finally, the modified sequence from the PacI site to ClaI was cloned into the Ad5max plasmid. K5, Ad5-knob; K37, Ad37-knob.

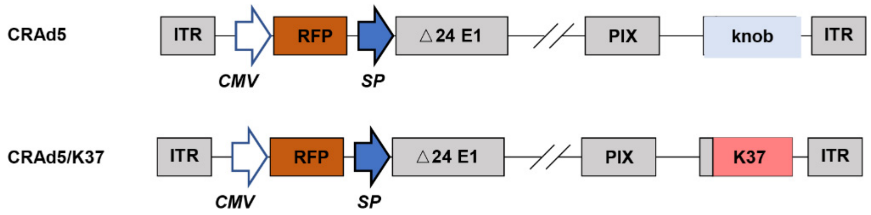

Figure S2

Genome of CRAd5 and CRAd5/K37. ITR, inverted terminal repeat; CMV, CMV promoter; RFP, red fluorescence protein; △24 E1, delta 24 E1; K37, Ad37-knob.

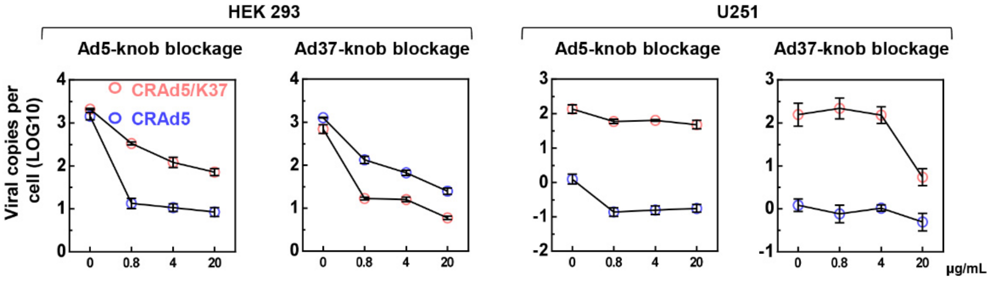

Figure S3

Virus binding quantification via soluble knob competition. Cells were harvested in tubes (5 × 105 per tube) and then incubated with soluble Ad5-knob or Ad37-knob at 4°C for 1 hour. After washing twice, adenoviruses were added at 104 MOIs for 1 hour-incubation at 4°C. The unbound viruses were removed by washing with PBS. Finally, the virus genome was extracted and quantified by qPCR for the number of viral copies bound on cells.

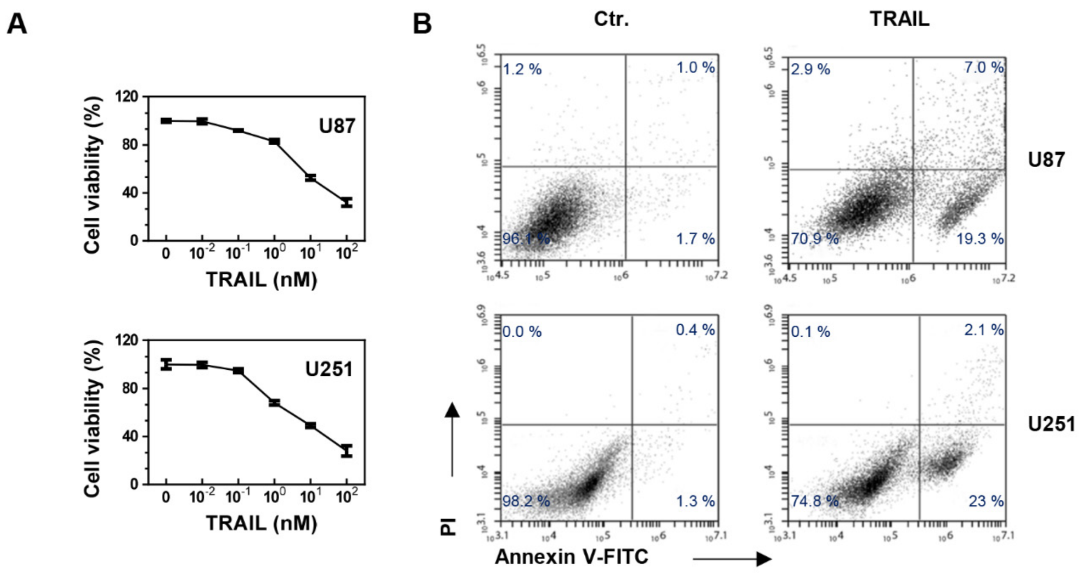

Figure S4

Sensitivity of glioma cells to TRAIL. A, Cell viability was analyzed by MTT assays, after glioma cells were incubated with soluble recombinant human TRAIL proteins for 16 hours. B, Cells were incubated with recombinant soluble human TRAIL protein (10 nM) for 6 hours at 37°C. Cell apoptosis was evaluated by annexin V-PI staining and subsequent FACS analysis.

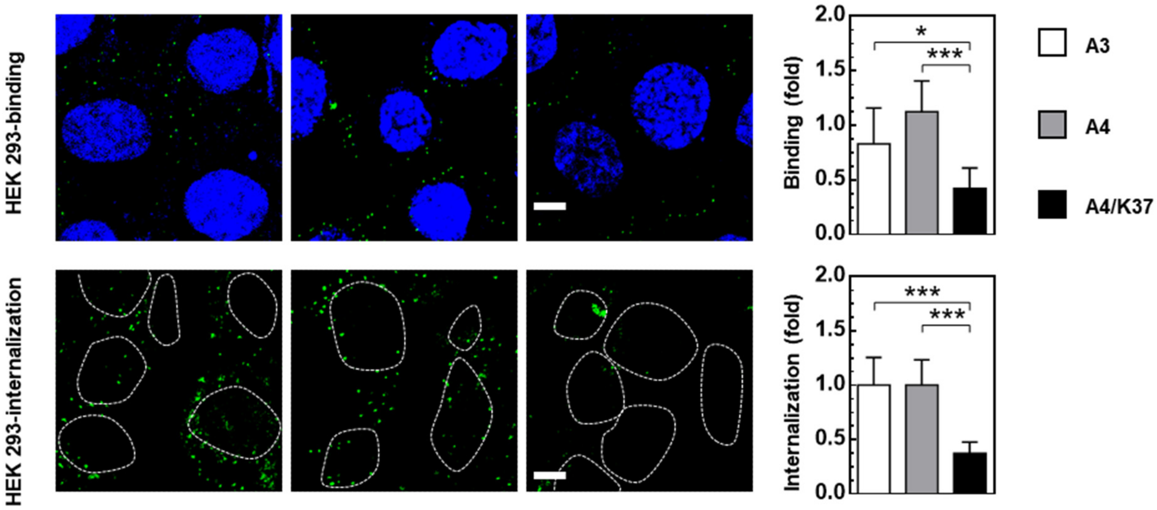

Figure S5

Virus binding and internalization in HEK 293 cells. Images show representative confocal stacks. Nuclei (DAPI stain) are blue and virus particles are green in the binding analysis; nuclei are shown as outlines in the images of internalization. Error bars were from values of three independent repeated experiments, and the significant differences between groups were analyzed using one-way ANOVA. *P < 0.05, ***P < 0.001.

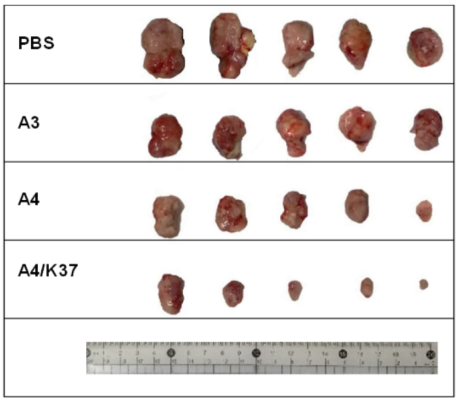

Figure S6

Tumor images. At the end of the experiment, tumors were dissected and photographed.

Supplementary Table

Comparison of some reported oncolytic adenoviruses and dual-modified oncolytic adenovirus in this study

	Virus	Capsid modification (s)	Receptor(s)	Therapeutic gene	Advantages	Outlook
**Reported oncolytic adenoviruses**	Onco-015 [1]	None	CAR	None	——	
	CRAd-Survivin-pk7 [2]	fiber modification with pk7 encoding polylysine	CAR, heparan sulfate receptor, polyanionic cellular receptor	None	■ Transformed infection mechanism improves infectivity and oncolytic efficacy towards glioma	
	Ad5/3 [3-5]	Fiber exchange with Ad3-knob	CD46, CD80/86	None		
	DNX2401 [6, 7]	RGD insertion in knob domain	CAR, integrin	None		
	H5CmTERT-Ad/TRAIL [8]	None	CAR	*TRAIL*	■ Mediate gene therapy	
	DNX2440 [9]	RGD insertion in knob domain	CAR, integrin	None (DNX2401), OX40L (DNX2440)	■ Transformed infection mechanism improves infectivity and oncolytic efficacy towards glioma<br>■ Gene therapy	
	Ad5/35-T [10]	Fiber exchange with Ad35-fiber	DSG2	*TRAIL*		
**Dual-modified oncolytic adenovirus in this study**	A4/K37	Fiber knob exchange with Ad37-knob<br>TRAIL modification on pIX	CAR, SA, TRAILRs	*TRAIL*	■ Transformed infection mechanism through 3 receptors improves infection and oncolytic effect towards glioma.<br>■ *TRAIl* gene mediated therapy	■ Therapeutic effects and the security need to be further evaluated in advanced models (e.g. PDX model).<br>■ The immunoregulatory effects for anti-glioma should be further evaluated.<br>…

References

1. Chiocca EA, Abbed KM, Tatter S, Louis DN, Hochberg FH, Barker F, et al. A phase I open-label, dose-escalation, multi-institutional trial of injection with an E1B-Attenuated adenovirus, ONYX-015, into the peritumoral region of recurrent malignant gliomas, in the adjuvant setting. Mol Ther. 2004; 10: 958-66.

2. Ulasov IV, Zhu ZB, Tyler MA, Han Y, Rivera AA, Khramtsov A, et al. Survivin-driven and fiber-modified oncolytic adenovirus exhibits potent antitumor activity in established intracranial glioma. Hum Gene Ther. 2007; 18: 589-602.

3. Guse K, Ranki T, Ala-Opas M, Bono P, Sarkioja M, Rajecki M, et al. Treatment of metastatic renal cancer with capsid-modified oncolytic adenoviruses. Mol Cancer Ther. 2007; 6: 2728-36.

4. Nandi S, Ulasov IV, Rolle CE, Han Y, Lesniak MS. A chimeric adenovirus with an Ad 3 fiber knob modification augments glioma virotherapy. J Gene Med. 2009; 11: 1005-11.

5. Nandi S, Ulasov IV, Rolle CE, Han Y, Lesniak MS. A chimeric adenovirus with an Ad 3 fiber knob modification augments glioma virotherapy. The journal of gene medicine. 2009; 11: 1005-11.

6. Lang FF, Conrad C, Gomez-Manzano C, Yung WKA, Sawaya R, Weinberg JS, et al. Phase I Study of DNX-2401 (Delta-24-RGD) Oncolytic Adenovirus: Replication and Immunotherapeutic Effects in Recurrent Malignant Glioma. J Clin Oncol. 2018; 36: 1419-27.

7. van den Bossche WBL, Kleijn A, Teunissen CE, Voerman JSA, Teodosio C, Noske DP, et al. Oncolytic virotherapy in glioblastoma patients induces a tumor macrophage phenotypic shift leading to an altered glioblastoma microenvironment. Neuro Oncol. 2018; 20: 1494-504.

8. Oh E, Hong J, Kwon OJ, Yun CO. A hypoxia- and telomerase-responsive oncolytic adenovirus expressing secretable trimeric TRAIL triggers tumour-specific apoptosis and promotes viral dispersion in TRAIL-resistant glioblastoma. Sci Rep. 2018; 8: 1420.

9. Jiang H, Rivera-Molina Y, Gomez-Manzano C, Clise-Dwyer K, Bover L, Vence LM, et al. Oncolytic Adenovirus and Tumor-Targeting Immune Modulatory Therapy Improve Autologous Cancer Vaccination. Cancer Res. 2017; 77: 3894-907.

10. Yang M, Yang CS, Guo WW, Tang JQ, Huang Q, Feng SX, et al. A novel fiber chimeric conditionally replicative adenovirus-Ad5/F35 for tumor therapy. Cancer Biol Ther. 2017; 18: 833-40.
